# Recent Updates on Molecular Imaging Reporting and Data Systems (MI-RADS) for Theranostic Radiotracers—Navigating Pitfalls of SSTR- and PSMA-Targeted PET/CT

**DOI:** 10.3390/jcm8071060

**Published:** 2019-07-19

**Authors:** Rudolf A. Werner, James T. Thackeray, Martin G. Pomper, Frank M. Bengel, Michael A. Gorin, Thorsten Derlin, Steven P. Rowe

**Affiliations:** 1 Department of Nuclear Medicine, Hannover Medical School, 30625 Hannover, Germany; 2 Johns Hopkins School of Medicine, The Russell H Morgan Department of Radiology and Radiological Science, Division of Nuclear Medicine and Molecular Imaging, Baltimore, MD 21287, USA; 3 The James Buchanan Brady Urological Institute and Department of Urology, Johns Hopkins University School of Medicine, Baltimore, MD 21287, USA

**Keywords:** positron emission tomography, PET, somatostatin receptor, SSTR, neuroendocrine tumors, NET, prostate carcinoma, prostate-specific membrane antigen, PSMA, theranostics, theragnostics, reporting and data system, RADS, molecular imaging reporting and data systems (MI-RADS), SSTR-RADS, PSMA-RADS

## Abstract

The theranostic concept represents a paradigmatic example of personalized treatment. It is based on the use of radiolabeled compounds which can be applied for both diagnostic molecular imaging and subsequent treatment, using different radionuclides for labelling. Clinically relevant examples include somatostatin receptor (SSTR)-targeted imaging and therapy for the treatment of neuroendocrine tumors (NET), as well as prostate-specific membrane antigen (PSMA)-targeted imaging and therapy for the treatment of prostate cancer (PC). As such, both classes of radiotracers can be used to triage patients for theranostic endoradiotherapy using positron emission tomography (PET). While interpreting PSMA- or SSTR-targeted PET/computed tomography scans, the reader has to navigate certain pitfalls, including (I.) varying normal biodistribution between different PSMA- and SSTR-targeting PET radiotracers, (II.) varying radiotracer uptake in numerous kinds of both benign and malignant lesions, and (III.) resulting false-positive and false-negative findings. Thus, two novel reporting and data system (RADS) classifications for PSMA- and SSTR-targeted PET imaging (PSMA- and SSTR-RADS) have been recently introduced under the umbrella term molecular imaging reporting and data systems (MI-RADS). Notably, PSMA- and SSTR-RADS are structured in a reciprocal fashion, i.e., if the reader is familiar with one system, the other system can readily be applied. Learning objectives of the present case-based review are as follows: (I.) the theranostic concept for the treatment of NET and PC will be briefly introduced, (II.) the most common pitfalls on PSMA- and SSTR-targeted PET/CT will be identified, (III.) the novel framework system for theranostic radiotracers (MI-RADS) will be explained, applied to complex clinical cases and recent studies in the field will be highlighted. Finally, current treatment strategies based on MI-RADS will be proposed, which will demonstrate how such a generalizable framework system truly paves the way for clinically meaningful molecular imaging-guided treatment of either PC or NET. Thus, beyond an introduction of MI-RADS, the present review aims to provide an update of recently published studies which have further validated the concept of structured reporting systems in the field of theranostics.

## 1. Theranostic Radiotracers for Neuroendocrine Tumors and Prostate Carcinoma

Theranostics is a paradigm-setting example of personalized medicine. It is based on the use of radiolabeled compounds which can be applied for both diagnostic molecular imaging and subsequent treatment, using different radionuclides for labelling. Theranostics has recently gained traction in clinical nuclear medicine departments, particularly in the diagnosis and treatment of neuroendocrine tumors (NET) and prostate carcinoma (PC) [1,2,3,4,5]. For the treatment of NET, membrane-bound somatostatin receptors (SSTR1, SSTR2, SSTR3, SSTR4, and SSTR5) on the cell surface enable targeted delivery of molecular diagnostic and therapeutic agents [5]. This concept has been exploited by diagnostic SSTR-targeted positron emission tomography (PET) radiotracers, such as ^68^Ga-labeled 1,4,7,10-tetraazacyclododecane-N,N′,N′′,N′′′-tetraacetic acid-d-Phe(1)-Tyr(3)-octreotide/-octreotate (^68^Ga-DOTATOC/-TATE). Once relevant SSTR expression has been confirmed at sites of disease, so-called “hot“ somatostatin analogs (SSA, ^177^Lu-DOTATATE/-TOC) can be administered in a therapeutic setting. After successful binding to the SSTR on the NET cell surface, these bound radiotracers are internalized into the cell and the release of ß-radiation from the internalized compound provokes DNA strand breaks [5]. Thus, peptide receptor radionuclide therapy (PRRT) resembles a Trojan horse mechanism, culminating in cellular demise [5]. As reported by the NETTER-1 trial, implementation of PRRT in the treatment algorithm of NET patients markedly improves outcome. Spearheaded by *Strosberg*, patients with well-differentiated metastatic midgut NET with unsuccessful first-line therapy (unlabeled “cold“ SSA) were randomized into either a control group receiving only high-dose SSA (octreotide long-acting repeatable (LAR) of 60 mg) or a treatment group of “hot SSA“ (^177^Lu-DOTATATE) plus octreotide LAR 30 mg. In this first randomized, controlled trial, the estimated progression-free survival at 20 months was 65.2% for the PRRT group (vs. the octreotide LAR group, 10.8%) with relatively negligible side effects. These findings were further corroborated, as the estimated risk of death was 60% lower for the ^177^Lu-DOTATATE arm [6]. In a recently published follow-up study, the time to health-related Quality of Life (QoL) deterioration, including global health status, physical and role functioning, disease-related mental stress, pain and diarrhea, was significantly longer in the ^177^Lu-DOTATATE group [7]. Thus, NETTER-1 emphasizes the high safety profile of the theranostic concept and a remarkable outcome benefit for patients suffering from metastatic midgut NET [5].

**Figure 1:**
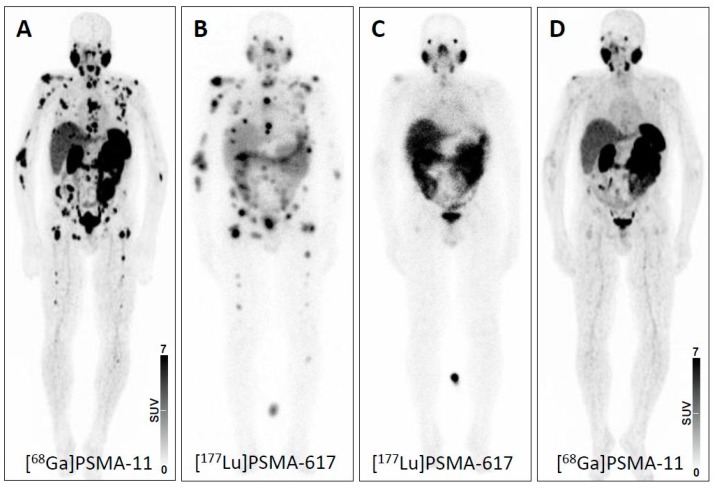
Theranostic concept in prostate carcinoma using prostate-membrane specific antigen (PSMA)-directed imaging and therapy. (**A**) PSMA-targeted positron emission tomography (PET) imaging before initiation of PSMA-targeted therapy showing widespread metastatic disease. Post-therapeutic scintigraphic scan after (**B**) one and (**C**) two cycles of PSMA-targeted therapy. (**D**) PSMA-targeted PET imaging after two cycles of therapy demonstrating high therapeutic efficacy and near-complete remission. Response was further corroborated by a decline in prostate specific antigen (PSA) level from 396.0 ng/mL to 7.35 ng/mL. PSMA: prostate-specific membrane antigen.

Recently, another theranostic approach using prostate-specific membrane antigen (PSMA)-targeted radiotracers for diagnostics and therapy of advanced prostate carcinoma (PC) has gained increased interest. This novel radiotracer addresses previously unmet clinical needs for PC including early detection, initial cancer staging and detection of local recurrence or metastases to guide appropriate therapy application, e.g., radioligand therapy (RLT) with its ^177^Lu-based counterpart [8]. Similar to PRRT for NET, recent reports have shown substantial benefit of PSMA-based RLT for end-stage PC patients with extensive tumor load, with a parallel decrease of tumor marker (prostate-specific antigen, PSA) levels [2]. Recently, 43 men suffering from metastatic castration-resistant PC were included in the single-arm, single-centre, prospective phase 2 LuPSMA trial. At time of study entry, all subjects had demonstrated progressive disease after first- and second-line treatments (including taxane-based chemotherapy and second-generation anti-androgens). After proving sufficient PSMA expression on the PC cells, individuals were scheduled for treatment with ^177^Lu-PSMA-617 and the majority of the subjects demonstrated an objective response rate of up to 82% in nodal or visceral disease. Notably, limited adverse effects were reported (Figure 1) [9].

The need for a standardized interpretation of oncologic imaging has been increasingly recognized, as it reduces variation in image interpretation [10] and subsequent clinical treatment [11], and assists in eliminating errors—all factors which have an incremental impact on patient safety [12,13]. Tracing its roots back to the 1970s, standardized reporting has been implemented more routinely in clinical medicine, e.g., by introducing the Système international d’unités (SI) units in laboratory medicine and widespread adoption of breast imaging reporting and data systems (BI-RADS) in mammography or Thyroid Imaging (TI)-RADS for thyroid ultrasound [14,15,16]. Given the evolving field of nuclear medicine with its increasing use of theranostic radiotracers, there is an indispensable need for generalizable framework systems for standardized reporting in the field [17]. Such framework systems would I) help convey to the nuclear medicine scan reader the level of certainty that an equivocal finding is a site of disease; II) help to navigate common pitfalls of either PSMA- or SSTR-PET/CT; III) facilitate communication with referring clinicians; IV) allow for comparison of results derived from multicenter studies; and V) identify appropriate candidates for treatment with ^177^Lu-labeled compounds in a theranostic setting [18].

Recently, two novel framework system, entitled PSMA-RADS (RADS) 1.0 for PSMA-PET/computed tomography (CT) and SSTR-RADS 1.0 for SSTR-PET/CT have been introduced [19,20]. Based on the same fundamental framework, these generalizable systems have been recently summarized under the umbrella term “Molecular Imaging RADS 1.0” (MI-RADS) [21]. Notably, MI-RADS can also be applied reciprocally, i.e., if a reader is familiar with one system, the other system can be readily understood [21].

**Figure 2:**
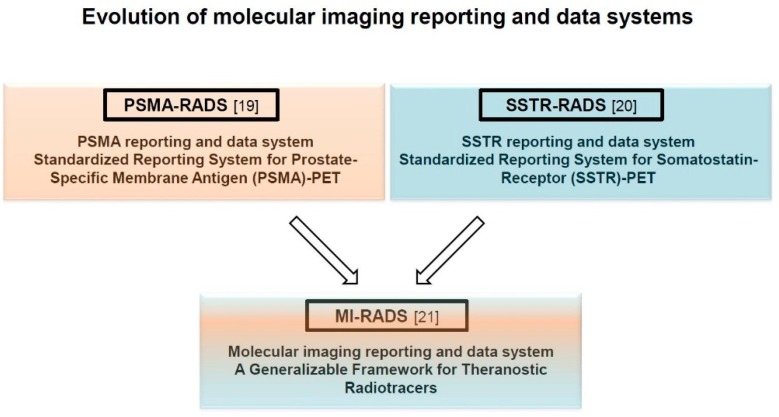
Evolution of molecular imaging reporting and data systems (MI-RADS). For theranostic radiotracers, two novel reporting and data system (RADS) classifications for PSMA- and SSTR-targeted PET imaging (PSMA- and SSTR-RADS) have been summarized under the umbrella term MI-RADS. SSTR: somatostatin receptor; RADS: reporting and data systems.

Here, we identify the most common pitfalls on PSMA- and SSTR-targeted PET/CT and present the recently-introduced novel framework system for theranostic radiotracers (MI-RADS, Figure 2) [21]. Finally, we apply MI-RADS to complex clinical cases, and, based on this framework system, propose molecular imaging-guided treatment strategies.

## 2. Common Pitfalls on SSTR- and PSMA-PET/CT

**Figure 3:**
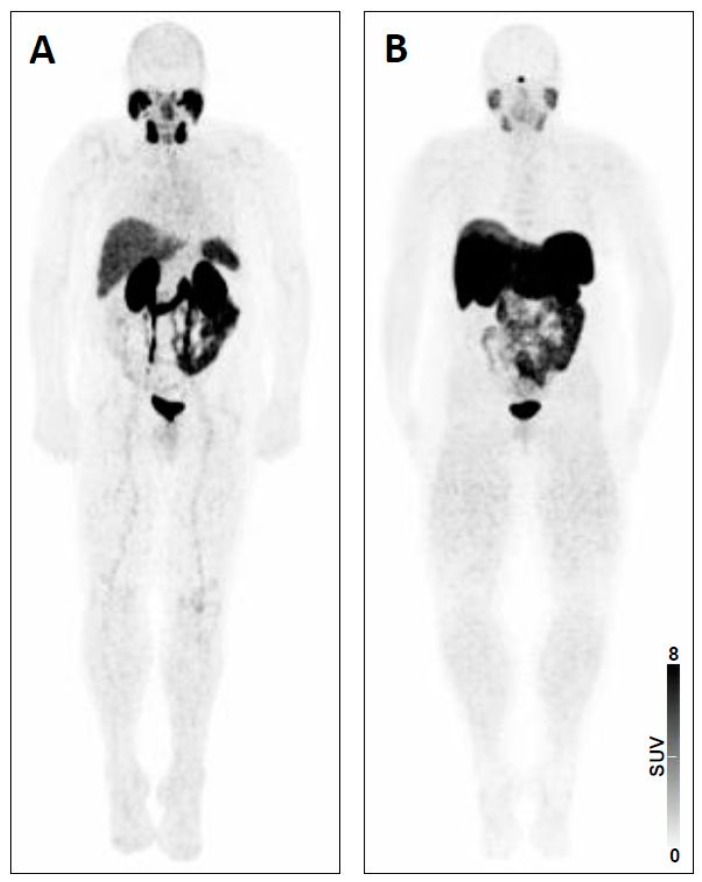
Whole-body maximum intensity projection images with normal biodistribution of (**A**) prostate-specific membrane antigen (PSMA)-targeted positron emission tomography (PET) using ^68^Ga-PSMA-11 and (**B**) somatostatin receptor (SSTR)-targeted PET using ^68^Ga-labeled 1,4,7,10-tetraazacyclododecane-N,N′,N′′,N′′′-tetraacetic acid-d-Phe(1)-Tyr(3)- octreotate (^68^Ga-DOTATATE).

Normal biodistribution on SSTR- and PSMA-PET/CT. The first step of interpreting a theranostic PET/CT is to ensure familiarity with the normal biodistribution of targeted tracers. For SSTR-PET, moderate to high uptake can be appreciated in pituitary gland, major salivary glands, thyroid, spleen (including splenosis, splenunculi), liver, both kidneys, both adrenal glands and uncinate process [22]. The latter finding on a normal SSTR-PET scan is important, as mistaking physiologic pancreatic uptake for sites of disease may lead to unnecessary surgical intervention, e.g., Whipple procedures [22]. For PSMA-PET, tracer accumulation can be observed in the lacrimal glands, salivary glands, liver, spleen, kidneys, small bowel and ganglia (with a descending frequency of PSMA signal in lumbar, cervical, stellate, celiac, and sacral ganglia) [19,23]. In addition, both radiotracers are excreted via the kidneys, clearly visualizing the urinary tract (Figure 3). However, normal tissue biodistribution differs among radiopharmaceuticals used for PSMA-PET imaging [24]. For instance, the ^18^F-labeled urea-based small-molecule PSMA inhibitor DCFPyL demonstrated slightly higher liver uptake compared to its ^68^Ga-labeled counterpart PSMA-11, whereas the latter agent exhibited increased radiotracer accumulation in the kidneys, spleen and major salivary glands [25]. Further complicating biodistribution, the novel ^18^F-labeled PSMA radiotracer ^18^F-PSMA-1007 displayed lower renal excretion; the resultant nonurinary excretion may prove valuable for delineation of local recurrence or small pelvic lymph node metastases (especially in close proximity to the ureters) [26]. Apart from physiological uptake pattern, recognition of potential sources of erroneous findings (either false-positive or false-negative) is essential for accurate interpretation of SSTR- and PSMA-PET/CTs [27].

**Figure 4:**
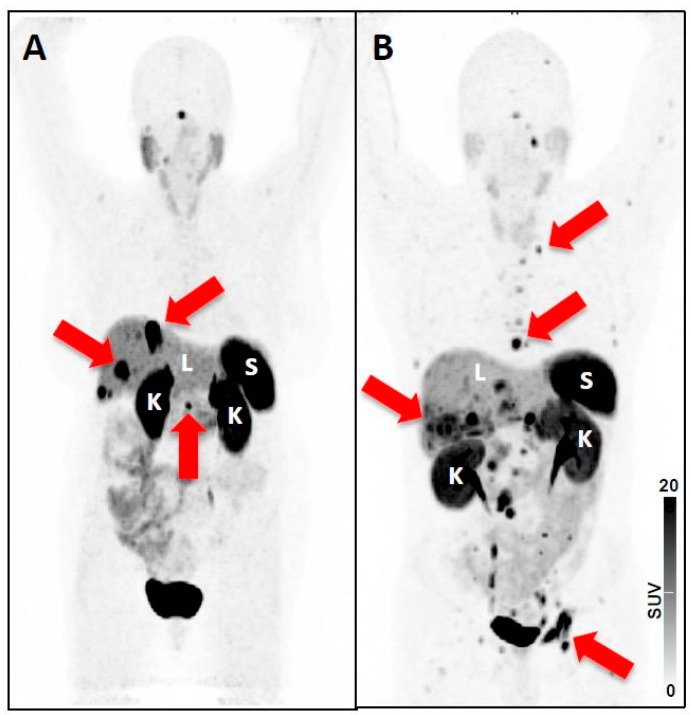
^68^Ga-DOTATATE maximum intensity projection (MIP) of patients with (**A**) low tumor burden, and (**B**) high tumor burden. Spleen (S), liver (L) and kidneys (K) are indicated, and the threshold has been set to a SUV of 15 in both scans. Red arrows indicate tumor lesions, which can be detected on the MIP: In Patient A SSTR-positive liver lesions and the SSTR-avid tail of the pancreas are highlighted. In Patient B several thoracic lymph node metastases are shown, but also several hepatic and bone lesions (e.g., in the acetabulum) can be detected. As ^68^Ga-DOTATATE has been used, a moderate tumor-sink effect can be appreciated, e.g. when comparing the kidneys of both patients [42]. Nonetheless, the visual assessed uptake in normal organs (visible for liver, kidneys, and spleen) does not differ substantially among the different patients. Thus, in patients with extensive tumor involvement, subtle lesions close to normal organs or other tumor lesions with high uptake can be missed, which in turn may decrease absolute lesion detection rate.

Pitfalls on SSTR-PET. The rising frequency of SSTR-PET to assess putative sites of disease in gastroenteropancreatic (GEP) NET patients has generated evidence of various benign and malignant conditions which may also have discernible radiotracer uptake. Such conditions include: (I) localized inflammation (e.g., prostatitis, post-radiation induced inflammation, large artery inflammation, atherosclerosis, culprit carotid lesions, (cardiac) sarcoidosis, myocardial infarction) [22,28,29,30,31,32,33,34]; (II) lesions of osteoblastic nature (e.g., degenerative structures, fracture, vertebral hemangioma) [20,22]; (III) other rare non-GEP NET tumors (medullary thyroid carcinoma) [35]; or (IV) incidental non-NET tumors (e.g., papillary thyroid carcinoma, follicular adenoma, non-Hodgkin lymphoma, breast cancer or meningioma) [5,36,37,38]. In addition, the impact of medication on non-invasive assessment of relevant SSTR expression should be considered. For instance, octreotide LAR diminished ^68^Ga-DOTATATE uptake in the liver, spleen, and thyroid, while the accumulation of the radiotracer remained unchanged in the primary tumor and metastatic lesions. Thus, scanning patients under octreotide LAR may even improve the lesion detection rate due to an enhanced tumor-to-background ratio [39]. These considerations are further fueled by a recent report also investigating long-acting SSA in patients scheduled for ^68^Ga-DOTATATE. Cherk et al. reported decreased uptake in normal organs but also increased radiotracer accumulation within metastases. Thus, interpretation of a ^68^Ga-DOTATATE PET/CT after treatment initiation with cold SSA requires extreme caution, as increased intensity alone may represent pseudoprogression rather than true progressive disease [40]. Nonetheless, further studies are warranted to determine the role of cold SSA on lesion uptake using SSTR-targeted PET/CT. Moreover, tumor burden can drastically affect normal organ biodistribution. As such, increased uptake in sites of disease can lead to a significant decrease in other organs, e.g., in a bone superscan with diminished renal uptake [41]. This concept also applies to SSTR-targeted agents, but radiotracer-specific variations must be considered. Specifically, ^68^Ga-DOTATATE exhibits a significant tumor sink effect in patients with variable of tumor loads (Figure 4) [42]. By contrast, ^68^Ga-DOTATOC displays no significant impact on normal organ biodistribution with increasing tumor burden, which may be explained by the higher affinity of ^68^Ga-DOTATATE for the SSTR2A receptor subtype (IC50 0.2 nM versus 2.5 nM for ^68^Ga-DOTATOC) [43,44]. Potential implications include increased dose to normal organs for subjects treated with ^177^Lu-DOTATOC, but also a decreased absolute lesion detection as compared to ^68^Ga-DOTATATE, particularly in challenging cases with high NET burden. Subtle uptake near sites of significant tumor burden, or normal organs with high expression levels of SSTR2, could be missed on a ^68^Ga-DOTATOC PET/CT. While such a scenario is likely rare, incorporation of structured reporting guidelines including a measure of uncertainty when characterizing lesions may guide the reader while interpreting such scans [44].

Pitfalls on PSMA-PET. Like SSTR-PET, manifold pitfalls have been described for PSMA-PET imaging, including a broad spectrum of either benign or malignant diseases associated with exaggerated uptake [27]. Several benign PSMA-avid pathologies can be misinterpreted as: (I) lymph node involvement (minimum one sympathetic ganglion demonstrating discernible radiotracer uptake in >97%) [23]; (II) bone lesions (bone remodeling, reparative processes including healing of fractures, Paget’s disease) [45,46,47]; (III) benign tumors of neurogenic origin (schwannoma, meningioma, paraganglioma, neurofibromas) [48,49,50]; (IV) benign vascular tumors (hepatic or subcutaneous capillary hemangioma) and tumor neovasculature [51,52]; (V) soft tissue lesions (gynecomastia, desmoid tumors, intramuscular myxoma, and pseudo-angiomatous stromal hyperplasia) [53,54,55,56]; or (VI) pulmonary involvement (granulomatous diseases (sarcoidosis, tuberculosis), anthracosilicosis, or chronic beryllium lung disease) [27,57,58,59,60]. However, non-prostatic malignant entities may also demonstrate relevant PSMA uptake, e.g., in the thyroid (medullary thyroid cancer), breast (triple-negative bilateral breast cancer), brain (glioblastoma multiform), lung (primary lung cancer) or in metastatic renal cell carcinoma [27,61,62,63,64]. Similar to SSTR-directed imaging, recent studies have also reported variable impact of tumor burden among commonly used PSMA-PET/CT radiopharmaceuticals. Gärtner and coworkers reported decreased renal ^68^Ga-PSMA-11 uptake in patients with higher tumor burden, whereas renal ^18^F-DCFPyL uptake was not similarly affected [65,66]. Again, such discrepant findings with different radiopharmaceuticals highlight the importance of structured reporting systems for patients with different tumor load, as lesions with moderate to faint uptake may be missed, especially close to a normal organ with exaggerated uptake.

## 3. Structured Reporting Systems for Theranostic Radiotracers—MI-RADS

### 3.1. MI-RADS 1.0. 

Given these pitfalls on both SSTR- and PSMA-PET/CT, readers may benefit from a structured reporting system while interpreting such scans. Consisting of PSMA- and SSTR-RADS, the MI-RADS frameworks are based on a five-point scale (from 1 = no evidence of disease and definitively benign to 5 = high certainty that either PC or NET are present), and indicate the site of disease and intensity of radiotracer uptake. Notably both RADS classification schemes for SSTR- and PSMA-PET/CT are designed in a reciprocal fashion, i.e., if the reader is familiar with one system, the other system can be readily applied. This design may further increase the feasibility to implement such a framework system into clinical practice [18,21].

### 3.2. SSTR-RADS 1.0. 

**Figure 5:**
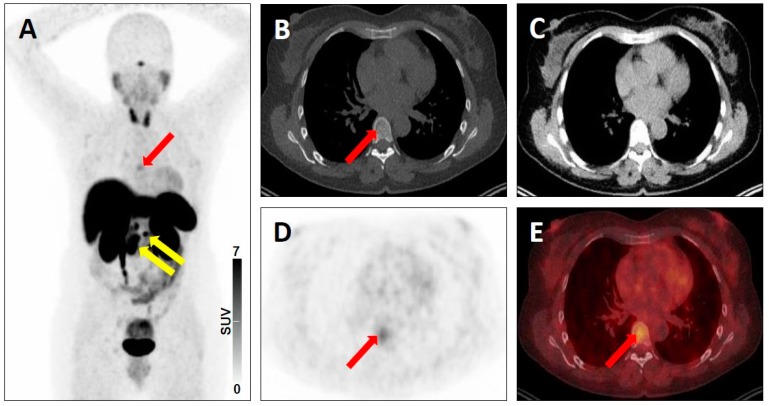
Equivocal uptake in bone lesion on SSTR-targeted positron emission tomography (PET)/computed tomography (CT). ^68^Ga-DOTATATE PET/CT for restaging in a 52-year-old woman with a history of ileal neuroendocrine carcinoma. Whole-body maximum intensity projection image showing multiple metastases to lymph nodes with clearly pathologic radiotracer uptake (yellow arrows), consistent with SSTR-RADS 5 lesions and a faint osteolytic bone lesion (red arrow) (**A**). Axial low-dose bone-window CT image (**B**), soft-tissue window CT image (**C**), PET image (**D**), and fused PET/CT image (**E**) further show this faint osteolytic bone lesion on anatomic imaging with equivocal uptake (red arrows), consistent with a SSTR-RADS-3B lesion.

**Figure 6:**
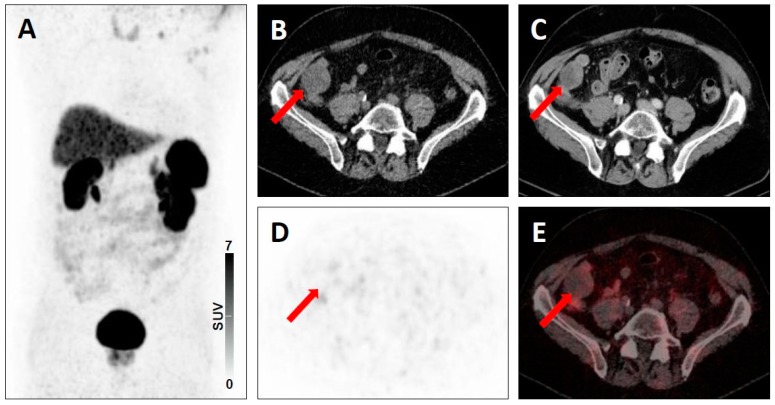
Lesion suggestive of malignancy on anatomic imaging but lacking uptake on SSTR-targeted positron emission tomography (PET)/computed tomography (CT). ^68^Ga-DOTATATE PET/CT for restaging in a 67-year-old man with a history of pancreatic neuroendocrine carcinoma. Whole-body maximum intensity projection image with no evidence of disease (**A**). Axial low-dose CT (**B**), contrast-enhanced CT (**C**), PET (**D**), and fused PET/CT (**E**) showing a peritoneal lesion suggestive of malignancy on anatomic imaging, but lacking uptake (red arrows), consistent with a SSTR-RADS-3D lesion.

SSTR-RADS-1 refers to benign lesions and is separated into the subcategories of SSTR-RADS-1A lesions, which include benign lesions characterized by either biopsy or anatomic imaging without any abnormal uptake and SSTR-RADS-1B which refers to similar sites with abnormal uptake (e.g., radiotracer-avid liver lesion, with magnetic resonance imaging (MRI) findings compatible with hemangioma). SSTR-RADS-2 lesions are likely benign and include those lesions, which have low-level uptake (i.e., ≤ blood pool level) and which are atypical for NET (e.g., uptake in a bone lesion strongly suggestive to be of degenerative nature). SSTR-RADS-2 differs from SSTR-RADS-1 in the certainty with which a malignant diagnosis can be excluded, with SSTR-RADS-2 representing those lesions where the possibility of a disease site exists, although it is remote (e.g., overlying uptake from a benign process could mask malignant uptake). SSTR-RADS-3 is the most complex category and is separated into four different subcategories. Notably, as it includes indeterminate lesions, further workup (e.g., biopsy or follow-up imaging) is often required. SSTR-RADS-3A describes equivocal uptake (approximately the level of blood pool) in a soft-tissue site typical for NET (e.g., a regional lymph node). SSTR-RADS-3B lesions include equivocal uptake in bone lesions not specifically atypical for NET on anatomic imaging. Follow-up imaging may confirm disease. While the first two SSTR-RADS-3 classifications have rather low-level uptake, SSTR-RADS-3C sites often have intense uptake, but in an atypical location for NET (e.g., an intense breast uptake on SSTR-PET). Biopsy would be recommended. SSTR-RADS-3D lesions do not have radiotracer uptake, but anatomic imaging raises suspicion of malignancy (e.g., a single liver lesion without SSTR expression but finding on conventional imaging). Biopsy of such lesions or follow-up imaging (e.g., by 2-deoxy-2-^18^F-fluoro-d-glucose) would be recommended. SSTR-RADS-4 describes those lesions with intense uptake in sites highly typical for NET, but lacking definitive evidence of disease on anatomic imaging, i.e., NET is highly likely. SSTR-RADS-4 and -5 differ in their correlate on conventional imaging: the latter classification also has intense radiotracer uptake, but corresponding findings can be appreciated on anatomic imaging modalities as well. NET is almost certainly present with SSTR-RADS-4/5 and PRRT is highly recommended for both SSTR-RADS-4/-5 categories [19,20,21,67]. Figure 5 and Figure 6 display two cases with SSTR-RADS-3B, -3D, and -5 lesions.

### 3.3. PSMA-RADS 1.0.

**Figure 7:**
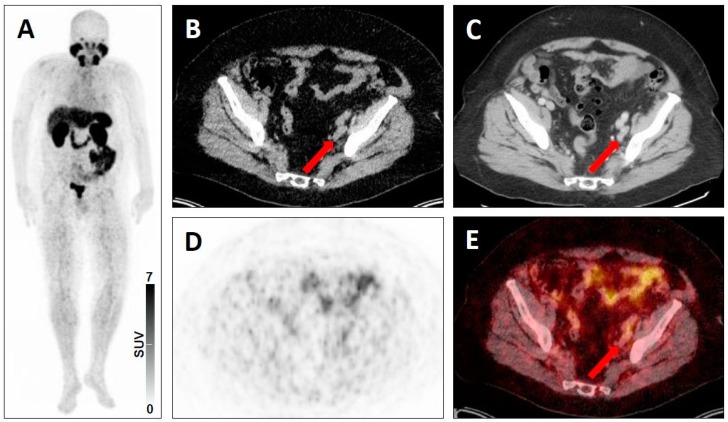
Equivocal uptake in soft-tissue lesion typical of prostate cancer lesion on PSMA-targeted positron emission tomography (PET)/computed tomography (CT). ^68^Ga-PSMA-11 PET/CT for restaging in a 73-year-old man with a history of a prostate cancer. Whole-body maximum intensity projection image showing absence of clearly pathologic radiotracer uptake (**A**). Axial low-dose CT (**B**), contrast-enhanced CT (**C**), PET (**D**), and fused PET/CT (**E**) showing an iliac lymph node typical of a prostate cancer lesion on anatomic imaging, but with equivocal uptake (red arrows), consistent with a PSMA-RADS-3A lesion.

**Figure 8:**
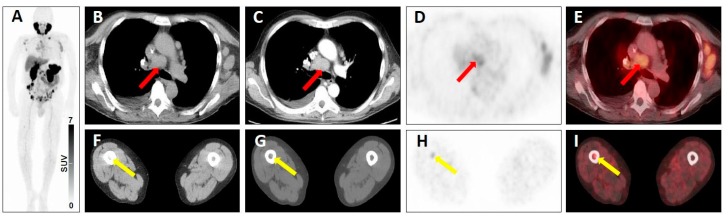
Equivocal uptake in soft-tissue and bone lesions on PSMA-targeted positron emission tomography (PET)/computed tomography (CT). ^68^Ga-PSMA-11 PET/CT for restaging in a 66-year-old man with a history of a prostate cancer. Whole-body maximum intensity projection image showing multiple metastases to lymph nodes and to the bone with both clearly pathologic radiotracer uptake and equivocal radiotracer uptake (**A**). Axial low-dose CT image (**B**), contrast-enhanced CT image (**C**), PET image (**D**), and fused PET/CT image (**E**) showing a mediastinal lymph node typical of a prostate cancer lesion on anatomic imaging, but with equivocal uptake (red arrows), consistent with a PSMA-RADS-3A lesion. Axial soft-tissue window CT image (**F**), bone-window CT image (**G**), PET image (H) and fused PET/CT image (I) showing a bone marrow lesion not atypical of a prostate cancer lesion on anatomic imaging, but with equivocal uptake (yellow arrows), consistent with a PSMA-RADS-3B lesion.

**Figure 9:**
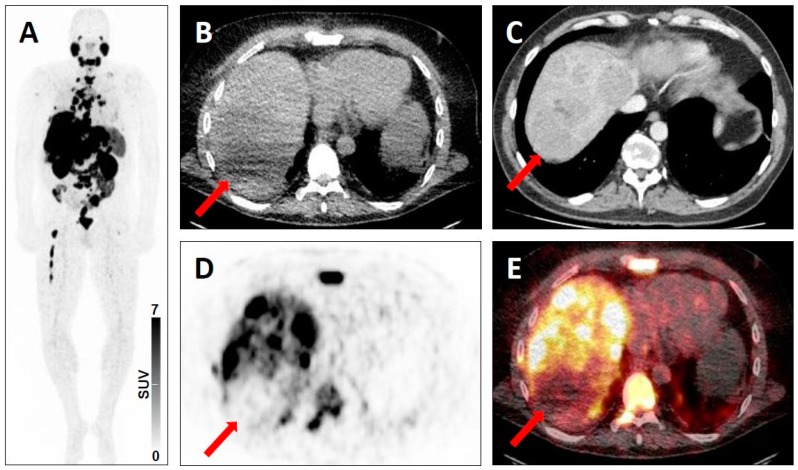
Lesion suggestive of malignancy on anatomic imaging but lacking uptake on PSMA-targeted positron emission tomography (PET)/computed tomography (CT). ^68^Ga-PSMA-11 PET/CT for restaging in a 52-year-old man with a history of a prostate cancer. Whole-body maximum intensity projection image demonstrating intense radiotracer uptake in metastases in the liver, lymph nodes and bone (**A**). Axial low-dose CT (**B**), contrast-enhanced CT (**C**), PET (**D**), and fused PET/CT (**E**) showing a hepatic lesion suggestive of malignancy on anatomic imaging, but lacking uptake (red arrows), consistent with a PSMA-RADS-3D lesion. In addition, there are numerous hepatic lesions with intense tracer uptake, consistent with a classification as PSMA-RADS-5 lesions.

As part of MI-RADS, PSMA-RADS also uses a five-point scale with higher numbers indicating a higher likelihood of malignancy. PSMA-RADS-1A refers to normal biodistribution and benign lesion (confirmed by biopsy or pathognomonic finding on anatomic cross-sectional imaging) without any abnormal uptake. PSMA-RADS-1B describes benign lesions but with abnormal radiotracer accumulation. PSMA-RADS 2 lesions show equivocal (focal, but ≤ blood pool level) uptake in soft tissue sites that would be atypical for metastatic PC (e.g., an axillary lymph node or an osteophyte). Similar to SSTR-RADS, PSMA-RADS-3 is also further divided into four subcategories. PSMA-RADS-3A refers to equivocal uptake in a soft tissue site that would be typical for metastatic PC (e.g., a pelvic lymph node involvement), while 3B refers to such lesions in the skeleton (equivocal uptake in a bone lesion typical in appearance on anatomic imaging for PC). Follow-up imaging, either with conventional or PSMA-PET/CT imaging may be recommended after three to six months to rule out malignancy. PSMA-RADS-3C describes intense radiotracer uptake in a site highly atypical for PC, except in end-stage disease (e.g., a PSMA-positive lung nodule in a patient with low level of serum PSA). PSMA-RADS-3D lesions demonstrate no discernible radiotracer uptake, but have an anatomic correlate on CT (e.g., PC of neuroendocrine origin), and these lesions should be further investigated, e.g., by a CT-driven biopsy. PSMA-RADS-4 and -5 are defined by intense uptake in typical sites of disease; however, for PSMA-RADS-4 lesions, no corresponding finding on anatomic imaging can be appreciated, while PSMA-RADS-5 describes lesions with intensive uptake and clear findings on CT. Similar to SSTR-RADS, PC is highly likely for the last two PSMA-RADS classifications and these patients should be scheduled for RLT [18,19,21]. Figure 7, Figure 8 and Figure 9 demonstrate the possible application of PSMA-RADS in clinic.

All MI-RADS classifications for PSMA-PET/CT (PSMA-RADS) and SSTR-PET/CT (SSTR-RADS) are summarized in Table 1 [18]. Table 2 lists common examples for all MI-RADS classification as described in the literature so far. For both MI-RADS systems, an overall (PSMA-) or (SSTR-)RADS score can be defined as well. The reader can identify up to five target lesions (most intense in uptake and largest in size). The overall RADS score is then defined as the highest scored target lesion among these five representative lesions. Thus, this highest-scored lesion takes priority and represents the overall scan impression. Specifically, if a scan is scored with a PSMA-RADS 3C and a PSMA-RADS 5 lesion, the overall PSMA-RADS score would be 5 (with further work-up recommendation for the PSMA-RADS 3C lesion) [21]. However, it remains a matter of debate whether treatment decisions for endoradiotherapy can be made based on the overall RADS score, i.e., on a single (but most dominant) lesion. Thus, future efforts should also turn towards integrating the entire tumor burden assessed with RADS to identify treatment candidates. For instance, a summed RADS score could be established, which takes the five target lesions of a scan into account. Further studies evaluating such concepts are needed to test MI-RADS in this scenario. Nonetheless, inclusion and exclusion criteria for endoradiotherapies based on established characteristics such as Ki67 in neuroendocrine tumors, and as-yet-not-completely-defined factors for PSMA-targeted therapy, still apply and should be considered [68,69].

Validation of MI-RADS. Prior to a more widespread adoption in clinical practice, framework systems such as MI-RADS should be more extensively validated. To date, studies evaluating SSTR-RADS are still lacking, and further research applying this system to SSTR-PET/CT are needed [18]. For PSMA-RADS, studies have focused on either reader agreement rates, or to determine the true nature of PSMA-RADS-3A and -3B lesions. Recently, the impact of PSMA-RADS for managing highly complex cases has also been reported [70,71,72].

**Figure 10:**
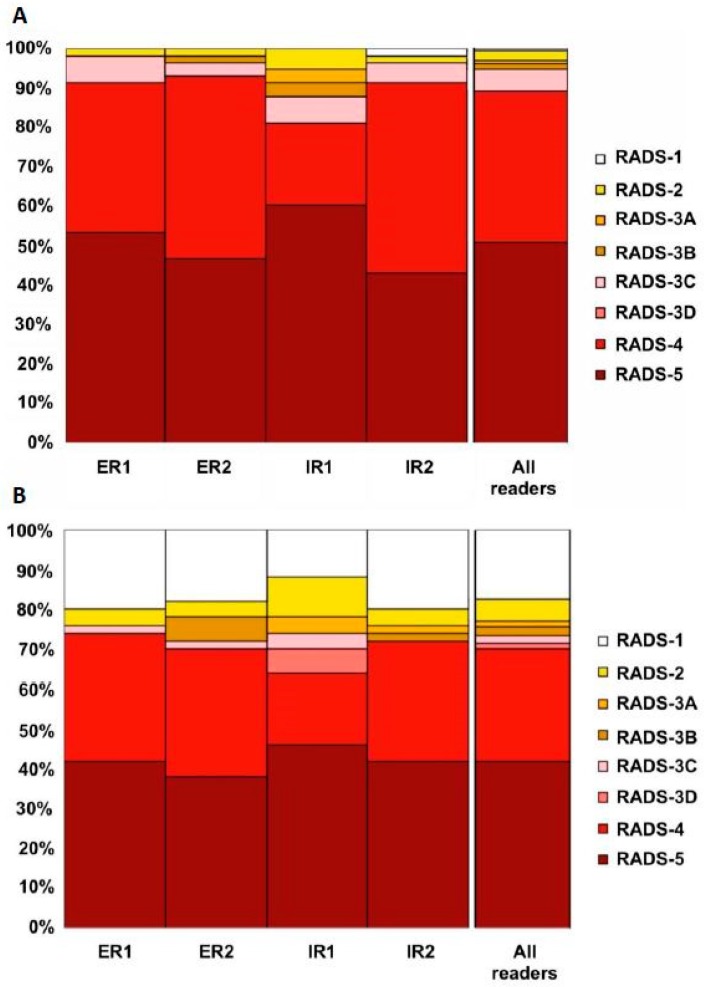
(**A**) Overview of target lesion (TL) assessment (identical target lesion included by all four readers on 50 PSMA-positron emission tomography (PET)/computed tomography (CT) scans with ^18^F-DCFPyL). The following organ compartments were defined: lymph nodes (LN), skeleton, prostate/local recurrence, soft tissue (other than LN), liver, thyroid, and lung. A PSMA-RADS Score had to be assigned to every target lesion by every blinded reader (ER, experienced reader, IR, inexperienced reader). Often, characterizing a lesion as PSMA-RADS-1B involves previous conventional imaging or histologic diagnosis; as such, PSMA-RADS-1A and -1B were subsumed under PSMA-RADS-1 in the present blinded interobserver agreement study. (**B**) Overview of overall PSMA RADS scoring for four blinded readers (ER, IR, assessment of 50 ^18^F-DCFPyL PET/CT scans). For the TL and overall scan impression, a high interreader agreement rate, even among IRs, was noted. Modified from Werner et al. [70], © by the Society of Nuclear Medicine and Molecular Imaging, Inc.

For PSMA-RADS, interobserver agreement was evaluated among four blinded readers with different experience [70]. In a prospective setting with 50 ^18^F-DCFPyL PET/CT scans, a maximum of 5 target lesions per scan were freely selected by the readers and a PSMA-RADS score for every target lesion was recorded. No specific preexisting conditions were placed on target lesion selection, although PSMA-RADS 1.0 suggests that readers focus on the most highly avid or largest lesions. An overall scan impression based on PSMA-RADS was indicated and interobserver agreement rates on a target lesion-based, organ-based, and overall PSMA-RADS score-based level were computed. The interobserver agreement for PSMA-RADS scoring among identical chosen target lesions was good for all readers. For lymph nodes and for an overall scan impression based on PSMA-RADS, excellent interobserver agreement was achieved (Figure 10). Thus, PSMA-RADS demonstrated a high concordance rate in this feasibility study, even among readers with different levels of experience. This suggests that PSMA-RADS can be effectively used for communication with clinicians and can be implemented in the collection of data for large prospective trials [70].

A recent longitudinal follow-up study investigated PSMA-RADS-3A (soft tissue) and PSMA-RADS-3B (bone) lesions which are defined as indeterminate for disease. The vast majority (75%) of PSMA-RADS-3A lesions exhibited changes with subsequent imaging, which were confirmed as malignancy. Conversely, among PSMA-RADS-3B indeterminate bone lesions, only 21.4% of cases exhibited altered tracer distribution suggestive of underlying PC. Notably, quantification approaches including the maximum standardized uptake value could not assess the true manifestation from a PSMA-PET scan, emphasizing the importance of this MI-RADS classification scheme for such indeterminate lesions [71].

In a recently published clinical use case with 18F-DCFPyL PET/CT, the PSMA-RADS grading system was applied to support decision making and treatment planning for a patient with PC. Due to high tumor load with widespread metastatic disease, a treatment plan was initiated utilizing the PSMA-RADS classification scheme, demonstrating the usefulness of a framework system to guide the clinician towards appropriate treatment on a lesion-by-lesion basis. Such an approach based on MI-RADS paves the way for molecular imaging-guided treatment of PC beyond endoradiotherapy [72].

## 4. Conclusions

Considering the manifold pitfalls of SSTR- and PSMA-PET/CT, a standardized framework system for radiotracers with theranostic implications, MI-RADS, is proposed. Similar to its predecessors in the radiology arena (BI-RADS, TI-RADS), the present framework system for molecular imaging should convey to the nuclear scan reader the level of certainty that an equivocal finding is a site of disease, identify and navigate common pitfalls and artefacts, facilitate communication with non-expert clinicians, and select appropriate candidates for treatment with ^177^Lu-labeled compounds in a theranostic setting. Efforts in the last year turned towards validating MI-RADS in a real-world scenario using the ^18^F-labeled compound DCFPyL for imaging in PC, including interobserver agreement studies, determining the true nature of lesions classified as indeterminate, and to test the practicality for challenging cases. In conclusion, MI-RADS provides the foundation to initiate molecular-imaging based treatment strategies on a lesion-by-lesion level, tailoring treatment to individual patient needs. There remain challenges to the implementation of such a system, particularly with regard to different normal biodistribution patterns and the variable impact of tumor burden among the available SSTR- and PSMA-PET radiopharmaceuticals, which should be addressed in future studies. The application for theranostic treatment selection remains to be fully tested. Nonetheless, the reciprocal design of MI-RADS makes the system easy to memorize and may lay the proper groundwork for a more widespread adoption of such framework systems into larger clinical trials for evaluating efficacy of SSTR-/PSMA-directed imaging and treatment.

## Figures and Tables

**Table 1:** Reporting and data systems (RADS) in molecular imaging (MI-RADS), consisting of prostate-membrane specific antigen (PSMA)-RADS and somatostatin receptor (SSTR)-RADS. n/a = not applicable, N(o) = peptide receptor radionuclide therapy (PRRT)/ radioligand therapy (RLT) not recommended. B = Biopsy, F/U = follow-up imaging (3–6 months, e.g. depending on Ki67 in NET). Y(es) = PRRT/RLT recommended. ^#^ = applies only to SSTR-RADS, defined as 1 (focal uptake, but ≤blood pool) through uptake level 2 (>blood pool, but ≤physiologic liver uptake) to uptake level 3 (>physiologic liver uptake). Modified from Werner et al. [21], original article has been distributed under the terms of the Creative Commons Attribution 4.0 International License (http://creativecommon-s.org/licenses/by/4.0/).

MI-RADS		PSMA- and SSTR-RADS	Workup	Uptake Level ^#^	PRRT/RLT?
1	1A	Benign lesion, characterized by biopsy or anatomic imaging *without* abnormal uptake	n/a	1	N
	1B	Benign lesion, characterized by biopsy or anatomic imaging *with* abnormal uptake	n/a	2–3	N
2		*Soft tissue* site or *bone* lesion *atypical* for metastatic PC or NET	n/a	1	N
3	3A	Equivocal uptake in *soft tissue lesion typical* of PC or NET	B, F/U	1–2	N
	3B	Equivocal uptake in *bone lesion not atypical* of PC or NET	B, F/U	1–2	N
	3C	Intense uptake in site *highly atypical* of all but advanced stages of PC or NET (i.e., high likelihood of nonprostatic/non-NET malignancy or other benign tumor)	B	3	N
	3D	Lesion *suggestive of malignancy* on anatomic imaging but *lacking uptake.* For SSTR-RADS: ^18^F-FDG is recommended to rule out potential dedifferentiation	B, F/U	not available	N
4		Intense uptake in site typical of PC or NET but *lacking* definitive findings on conventional imaging	n/a	3	Y
5		Intense uptake in site typical of PC or NET and *with* definitive findings on conventional imaging	n/a	3	Y

**Table 2:** Common examples for all classifications, as described in the literature to date. Reporting and data systems (RADS) in molecular imaging (MI-RADS), somatostatin receptor (SSTR)-RADS, and prostate-membrane specific antigen (PSMA)-RADS.

MI-RADS		Examples on SSTR-PET/CT	Examples on PSMA-PET/CT
1	1A	Normal physiologic biodistribution of an SSTR imaging agent [20]	Normal physiologic biodistribution of a PSMA imaging agent [19]
	1B	- Prostatitis [20] - Benign prostatic hyperplasia [20] - Radiotracer-avid liver lesion, with magnetic resonance imaging findings compatible with hemangioma [20]	- Thyroid nodules with uptake that have been previously biopsied and found to be benign [19] - Liver hemangioma with focal uptake that have been characterized with liver protocol CT or MRI [19] - Adrenal adenoma with radiotracer uptake with characteristic conventional imaging findings [19]
2		- Axillary lymph nodes [20] - Schmorl’s node in a vertebral body [20]	- Isolated mediastinal or axillary lymph nodes with minimal uptake, healing fractures [19] - Focal splenic uptake [21]
3	3A	- Regional lymph nodes, e.g. low-level uptake in mesenteric lymph node in midabdomen [20] - Mild radiotracer uptake in a supraclavicular lymph node [5]	- Pelvic lymph node involvement with low-level uptake [19]
	3B	- Low-level uptake in a rib with lack of anatomic correlate [20]	- Low level-uptake in the iliac bone with lack of anatomic correlate [19]
	3C	- Intense breast uptake on SSTR-PET [20,21] - Intense uptake in the Musculus vastus lateralis [18]	- High level of radiotracer uptake in a lung nodule in a patient with low level of serum prostate-specific antigen [19] - Substernal thyroid nodule with radiotracer uptake (and without further work-up) [21,72]
	3D	- Single liver lesion without SSTR expression but finding on conventional imaging [20] - Modest/No radiotracer uptake in the primary of the lung, with intense radiotracer uptake on 2-deoxy-2-18F-fluoro-d-glucose PET two weeks later [18]	- Prostate cancer (PC) of neuroendocrine origin [19] - Non-radiotracer-avid lung nodule in a patient with biochemically recurrent PC [19]
4		- Intense uptake in a liver lesion without definitive findings on conventional imaging [20]	- Intense radiotracer uptake in a lymph node without definitive findings on conventional imaging [19]
5		- Intense uptake in a liver lesion with definitive findings on conventional imaging [20]	- Extensive metastatic PC with diffuse osseous metastatic disease and intense radiotracer uptake (“superscan“ on PSMA-PET/CT) [19]

Common examples for all classifications, as described in the literature to date. Reporting and data systems (RADS) in molecular imaging (MI-RADS), somatostatin receptor (SSTR)-RADS, prostate-membrane specific antigen (PSMA)-RADS.
